# Molecular characteristics and clinical outcomes of complex *ALK* rearrangements identified by next-generation sequencing in non-small cell lung cancers

**DOI:** 10.1186/s12967-021-02982-4

**Published:** 2021-07-16

**Authors:** Peiyi Xia, Lan Zhang, Pan Li, Enjie Liu, Wencai Li, Jianying Zhang, Hui Li, Xiaoxing Su, Guozhong Jiang

**Affiliations:** 1 Department of Pathology, The First Affiliated Hospital of Zhengzhou University, Zhengzhou University, Jian She Dong Road 1, Zhengzhou, 450052 Henan China; 2 Institute of Medical and Pharmaceutical Sciences, Zhengzhou University, Zhengzhou, 450052 China; 3 Clinical Research Division, Berry Oncology Corporation, Fuzhou, 350200 China

**Keywords:** *ALK* fusion, Complex rearrangements, Non-small cell lung cancer, Next-generation sequencing, Targeted therapy

## Abstract

**Background:**

Complex kinase rearrangement, a mutational process involving one or two chromosomes with clustered rearrangement breakpoints, interferes with the accurate detection of kinase fusions by DNA-based next-generation sequencing (NGS). We investigated the characteristics of complex *ALK* rearrangements in non-small cell lung cancers using multiple molecular tests.

**Methods:**

Samples of non-small cell lung cancer patients were analyzed by targeted-capture DNA-based NGS with probes tilling the selected intronic regions of fusion partner genes, RNA-based NGS, RT-PCR, immunohistochemistry (IHC) and fluorescence in situ hybridization (FISH).

**Results:**

In a large cohort of 6576 non-small cell lung cancer patients, 343 (5.2%) cases harboring *ALK* rearrangements were identified. Fourteen cases with complex *ALK* rearrangements were identified by DNA-based NGS and classified into three types by integrating various genomic features, including intergenic (n = 3), intragenic (n = 5) and “bridge joint” rearrangements (n = 6). All thirteen cases with sufficient samples actually expressed canonical *EML4-ALK* fusion transcripts confirmed by RNA-based NGS. Besides, positive ALK IHC was detected in 13 of 13 cases, and 9 of 11 cases were positive in FISH testing. Patients with complex *ALK* rearrangements who received *ALK* inhibitors treatment (n = 6), showed no difference in progression-free survival (PFS) compared with patients with canonical *ALK* fusions n = 36, *P* = 0.9291).

**Conclusions:**

This study firstly reveals the molecular characteristics and clinical outcomes of complex *ALK* rearrangements in NSCLC, sensitive to ALK inhibitors treatment, and highlights the importance of utilizing probes tilling the selected intronic regions of fusion partner genes in DNA-based NGS for accurate fusion detection. RNA and protein level assay may be critical in validating the function of complex *ALK* rearrangements in clinical practice for optimal treatment decision.

**Supplementary Information:**

The online version contains supplementary material available at 10.1186/s12967-021-02982-4.

## Background

Rearrangements of the anaplastic lymphoma kinase (*ALK*) gene have been identified in approximately 3–7% of non-small cell lung cancer (NSCLC) patients, with echinoderm microtubule-associated protein like-4 (*EML4*) representing the most common fusion partner [1, 2]. *ALK*-rearranged NSCLC define a distinct molecular subset with high sensitivity to *ALK* tyrosine kinase inhibitors (TKIs). Crizotinib, a well-tolerated first generation *ALK* inhibitor [3, 4], has been approved by Food and Drug Administration in US for the treatment of *ALK*-rearranged NSCLC in 2011. Second generation *ALK* inhibitors, such as alectinib and ceritinib, are effective not only in crizotinib-naive patients [5], but also in patients with acquired resistance to crizotinib [6–9]. The identification of *ALK* rearrangements and the approval of a number of *ALK* TKIs have revolutionized the treatment of patients harboring *ALK* fusions. Therefore, accurate detection for *ALK* rearrangements is crucial.

A challenge for precision oncology is identifying novel or complex translocations. Traditional methods, including fluorescence in situ hybridization (FISH) and immunohistochemistry (IHC), have limitations, such as FISH does not permit identification of *ALK* partner genes or non-canonical breakpoints, and ALK IHC could be confounded in principle by overexpression of *ALK* driver rather than a true fusion protein [10]. While next-generation sequencing (NGS) techniques provide an effective and accurate detection for known and novel oncogenic fusions, and have been widely applied in clinical diagnostics.

Complex kinase rearrangements, herein referred to a mutational process involving one or two chromosomes with clustered rearrangement breakpoints. Recent studies have revealed that complex genomic rearrangements generated 74% of known fusion oncogenes in human lung adenocarcinoma of non-smokers, including *EML4-ALK*, *CD74-ROS1*, and *KIF5B-RET* [11]. However, complex genomic rearrangements frequently hindered proper capture in DNA-based NGS assay [12]. Accumulating evidences have suggested that genomic breakpoints identified by DNA sequencing are an unreliable predictor of breakpoint at the transcript level owing to genomic complexities [13, 14]. The identification and clinically functional validation of complex kinase rearrangements remain elusive, which makes the oncologists confused to choose the appropriate treatments. A combination methodology of DNA-based NGS technique followed by RNA-based NGS provides a unique opportunity to explore the mutational processes in cancer genomes. Although there has been landmark study characterizing the complex intergenic-breakpoint fusions [14], it was largely based on exome and selected introns in *ALK* gene. It is lack of the study using DNA-based NGS designed for intronic regions from fusion partner genes known to likely harbor the genomic breakpoint.

Herein, we utilized DNA-based NGS panel specifically designed with multiple probes tilling selected intronic regions of fusion partner genes, and identified three types of complex *ALK* rearrangements in 14 cases from a large cohort of NSCLC patients. Further functional validation performed by RNA or protein assay elucidated the importance of DNA and RNA-based NGS for the comprehensive detection of kinase fusions and guiding optimal treatment decision.

## Methods

### Patients and samples

Samples from a cohort of 6576 patients with NSCLC from January 2018 to July 2020 were collected for molecular testing. Pathological and clinical information was obtained from clinical records. The study was approved by the Institutional Review Board of the First Affiliated Hospital of Zhengzhou University. All patients provided informed written consent for these genomic analyses.

### DNA/RNA extraction

The pathological diagnosis of each case was confirmed on routine hematoxylin and eosin stained slides, and the corresponding optimal blocks containing a minimum of 20% tumor cells were forwarded for DNA/RNA extraction. Genomic DNA (gDNA) and total RNA were extracted from the formalin-fixed paraffin-embedded (FFPE) tumor tissue samples using AllPrep DNA/RNA FFPE Kit (Qiagen, USA) according to the manufacturer’s instructions. As a control, gDNA from the white blood cell samples was extracted using MagPure Blood DNA DA Kit (Magen, China) according to the manufacturer’s instructions. The quality of purified DNA/RNA were assayed by gel electrophoresis and quantified by Qubit® 4.0 Fluorometer (Life Technologies, USA). The amounts of extracted DNA more than 30 ng were considered sufficient for analysis. In the extracted FFPE RNA samples, the 28S and 18S rRNA bands were degraded, and ≥ 200 ng RNA were optimal for high analytical sensitivity.

### DNA-based NGS

The purified gDNA was first fragmented into DNA pieces about 300-bp using enzymatic method (5X WGS Fragmentation Mix, Qiagen, USA), followed by end repairing, T-adaptors ligation, and PCR amplification, resulting in pre-library. An in-house designed panel targeting most exons and selected introns in 86 cancer-related genes was used to capture DNA fragments to detect SNV/Indel, copy number variation and gene fusions (Additional file 1: Table S1). Particularly, hybrid capture-probes tilling the intronic regions of *ALK* (intron 18–19), *EML4* (intron 6, 13, 20) and *KIF5B* (intron 15–16, 24) were designed for the detection of *ALK* rearrangements event. Sequencing libraries were generated after PCR amplification and then sequenced on NovaSeq 6000 platform (Illumina, San Diego, USA) with 150PE mode.

Initial read mapping against the human reference genome hg19 and alignment processing was performed using BWA [15]. SAMtools [16] and Genome Analysis Toolkit GATK 3.8 [17] were used to call SNVs and small indel variants. Large indels and chromosomal rearrangements (including *ALK* rearrangements) were analysed using Fusionmap [18]. The nonsynonymous SNVs with VAF > 0.5% or with VAF > 0.1% in cancer hotspots collected from patient database were kept for the further analysis. Fusions with coverage ≥ 300 and supported mutation reads number ≥ 3 were identified and reported. For breakpoints in intergenic regions, the nearest gene in each direction was reported as the predicted fusion partner.

### RNA-based NGS

An in-house designed RNA fusion panel based on hybrid capture sequencing (Berry Oncology Corporation) was performed to detect gene fusions, which tilling all coding exons of common fusion genes in cancer and allowing for detection of known and novel fusions without a limitation for fusion partner or breakpoint. Briefly, the purified total RNA was first converted to complimentary DNA (cDNA) through reverse transcription reaction. The pre-libraries construction consisted of end repairing, adaptor ligation and PCR amplification, which of the total amounts was optimized for a desired value of ≥ 600 ng. The follow-up hybridization-captured libraries were sequenced on NovaSeq 6000 platform (Illumina, San Diego, USA) with paired-end 150-bp reads. Gene fusions were called based on Fusionmap software [18]. Bioinformatically identified fusions were verified by manual inspection of the breakpoints.

### FISH

In brief, FFPE tumor tissue samples was analyzed by FISH using the Vysis LSI ALK Dual color, Break Apart Rearrangement Probe (Abbott/Vysis, Abbott Park, IL, USA). In 50 scored tumour cells of every sample, if more than 15% of the scored tumour cells had split one or both ALK 5′ and 3′ probe signals or had isolated 3′ signals, the sample was considered to be FISH positive. Every FISH slide was evaluated by two pathologists independently.

### IHC

Immunohistochemistry of ALK protein was performed on a fully automated Ventana Benchmark XT stainer (Ventana Medical Systems, Roche Group, Tucson, AZ). FFPE tumor samples were stained using the pre-diluted Ventana anti-ALK (D5F3) Rabbit monoclonal primary antibody and a matched Rabbit Monoclonal Negative Control Ig antibody, together with the Optiview DAB IHC detection kit and Optiview Amplification kit. Every IHC slide was evaluated by two pathologists independently. Neoplastic cells labeled with the ALK IHC assay are evaluated for presence or absence of the DAB signal according to the method previously described [19]. If strong granular cytoplasmic staining was observed in any tumor cells at any percentage, the sample was considered to be ALK positive, while the sample without strong granular cytoplasmic staining in tumor cells was considered to be ALK negative.

### Clinical response evaluation and statistical analysis

For a subset of patients who received targeted *ALK* inhibitors treatment, clinical responses were assessed based on computed tomography (CT) imaging, following the Response Evaluation Criteria in Solid Tumors (RECIST) version 1.1. The association of patient characteristics and clinicopathological factors was investigated by the chi-square test. Progression-free survival (PFS) was calculated using the Kaplan–Meier method and differences in variables using the log-rank test. A two-sided *P* < 0.05 was considered to be statistically significant. Statistics were analyzed using GraphPad Prism (version 7.04).

## Results

### Characteristics of patients and *ALK* rearrangements

**Fig. 1:**
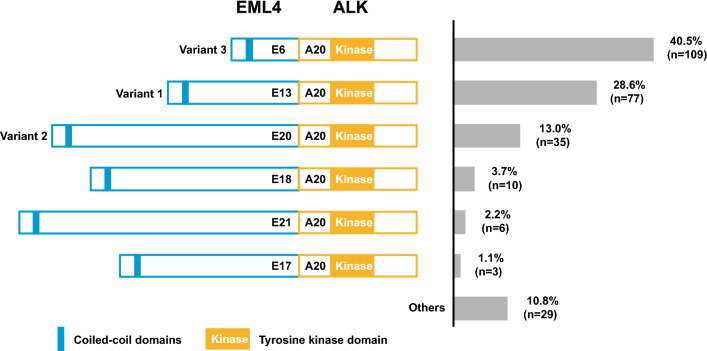
Schematic diagrams and distribution frequency of *EML4-ALK* fusion variants in the study cohort (N = 269). Abbreviation: E: *EML4* exon; A: *ALK* exon

All of 6576 samples from NSCLC patients were profiled with DNA-based NGS between January 2018 and July 2020. The clinical characteristics of the patients are described in Table 1. *ALK* fusions were identified in 343 (5.2%) cases with higher incidences in female, age < 60 or adenocarcinoma patients. Canonical *EML4-ALK* fusions occurred most frequently accounting for 78.4% (269/343). Most of the genomic breakpoints of the *ALK* gene were detected within intron19, while the *EML4* potential breakpoints differ and may generate various fusion protein variants at the genomic level. As shown in Fig. 1, *EML4-ALK* variant 3 (E6:A20, 109/269, 40.5%) was the most predominant type, followed by variant 1 (E13:A20, 77/269, 28.6%) and variant 2 (E20:A20, 35/269, 13.0%).

**Table 1:** Characteristics of NSCLC patients subjected to DNA-based NGS

Patient	Total no	ALK Gene fusions	P
Gender			
Male	3699	166 (4.5%)	0.002601
Female	2877	177 (6.2%)	
Age			
≥ 60	3704	107 (2.9%)	< 0.001
< 60	2872	236 (8.2%)	
Histology			
Adenocarcinoma	4897	311 (6.4%)	< 0.001
Squamous	982	7 (0.7%)	
Other*	697	25 (3.6%)	
Sample type			
Surgical	1689	98 (5.8%)	0.3983
Biopsy/cell block	4571	239 (5.2%)	
Liquid biopsy	316	6 (1.9%)	

^*^Other carcinomas included adenosquamous carcinoma (n = 76), large cell cancer (n = 17), large cell neuroendocrine carcinoma (n = 25), neuroendocrine carcinoma (n = 22), sarcomatoid carcinoma (n = 36), non-small cell lung cancer-not otherwise specified (n = 287) and unknown (n = 234)

### Identification and validation of complex *ALK* rearrangements

Among the 343 *ALK* fusion cases, complex *ALK* rearrangements in 14 cases were identified using targeted DNA-based NGS across 86 cancer-related genes panel with multiple probes tilling selected intronic regions of fusion partner genes (Table 2). These cases could be divided into three types by integrating various genomic features, including intergenic (n = 3), intragenic (n = 5) and “bridge joint” rearrangements (n = 6). A subset of 13 cases retained enough specimens were validated for additional RNA-based NGS tilling all coding exons of common fusion genes. Surprisingly, we found that the fusion genes and breakpoint positions had significant discrepancies between DNA and RNA sequencing. All thirteen cases actually expressed canonical *EML4-ALK* fusion transcripts. Besides, positive ALK IHC was detected in 13 of 13 cases, and 9 of 11 cases were positive in FISH testing.

**Table 2:** Comparison of DNA-based NGS, RNA-based NGS, IHC and FISH results among 14 lung cancer cases

Case	Sex	Age	Diagnosis	DNA-based NGS	RNA-based NGS	IHC	FISH
1	M	54	ADC	*EML4-intergenic* (intron13: intergenic) *WDR43-ALK* (3′ intron1: 3′ intron19)	*EML4-ALK* (exon13: exon20)	ALK+	N/A
2	F	32	ADC	*intergenic-EML4* (intergenic: intron6) *ALS2CR11-ALK* (3′ intron9: 3’intron19) *ALK-RBKS* (intron19: intron5)	*EML4-ALK* (exon6: exon20)	ALK+	+
3	M	45	ADC	*EML4-intergenic* (intron6: intergenic) *PLXNA4-ALK* (3′ intron3: 3′ intron19) *intergenic-EML4* (intergenic: intron6)	*EML4-ALK* (exon6: exon20)	ALK+	+
4	M	28	ADC	*EML4-ALK* (intron13: intron3) *ALK-ALK* (intron3ins9: intron19) *GALM-EML4* (intron4: intron13) *ALK-intergenic* (intron19: intergenic)	*EML4-ALK* (exon13: exon20)	ALK+	+
5	M	63	ADC	*EML4-ALK* (5′ intron6: 5′ intron19) *ALK-ALK* (3′ intron18: 3′ intron19) *IL1RAPL2-ALK* (5′ intron6: 5′ intron18) *ALK-PTCHD1* (3′ intron18: 3′ intron1)	*EML4-ALK* (exon6: exon20)	ALK+	+
6	F	54	ADC	*EML4-ALK* (intron6ins39: intron4) *ALK-ALK* (intron4ins57: intron19)	N/A	N/A	N/A
7	F	54	ADC	*EML4-EML4* (5′ intron6: 5′ intron6) *EML4-ALK* (3′ intron6: 3′ intron19)	*EML4-ALK* (exon6: exon20) *EML4-ALK* (exon6ins33: exon20)	ALK+	+
8	M	50	ADC	*EML4-EML4* (5′ intron6: 5′ intron6) *EML4-ALK* (3′ intron6ins4: 3′ intron19)	*EML4-ALK* (exon6: exon20)	ALK+	+
9	M	75	ADC	*EML4-LCLAT1* (5′ intron13: 5′ intron1) *LCLAT1-ALK* (3′ intron1: 3′ intron19)	*EML4-ALK* (exon13: exon20)	ALK+	−
10	M	59	ADC	*EML4-NEB* (intron13: intron24) *NEB-ALK* (intron24: intron19)	*EML4-ALK* (exon13: exon20)	ALK+	+
11	F	44	ADC	*EML4-KCNQ3* (intron6: intron1) *KCNQ3-ALK* (intron1: intron19) *ALK-EML4* (intron19: intron6)	*EML4-ALK* (exon6: exon20)	ALK+	+
12	F	43	ADC	*EML4-RUNX1* (intron5: intron5) *RUNX1-ALK* (intron6: intron19)	*EML4-ALK* (exon5: exon20) *RUNX1-ALK* (exon6: exon20)	ALK+	−
13	M	56	ADC	*EML4-ZNF362* (intron13: intron1) *ZNF362-ALK* (intron1: intron19)	*EML4-ALK* (exon13: exon20)	ALK+	N/A
14	M	31	ADC	*EML4-RPIA* (intron13: intron3) *MAP4K3-ALK* (intron2: intron19)	*EML4-ALK* (exon13: exon20)	ALK+	+

F: female; M: male; ADC: adenocarcinoma; N/A: not available; +: positive results; −: negative results

**Fig. 2:**
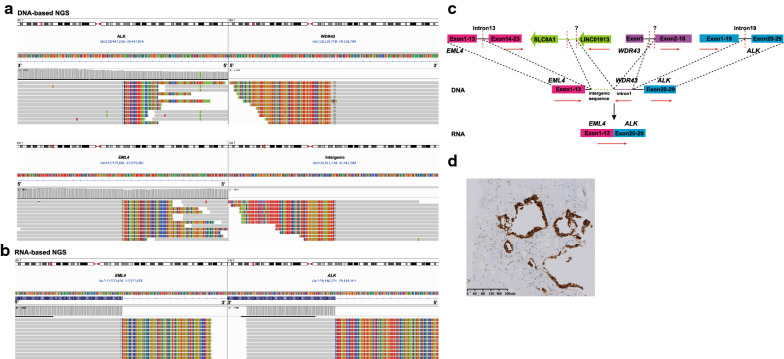
Representative example of intergenic complex rearrangements generating *EML4-ALK* fusion transcripts from case 1. **A** DNA sequencing reads indicating *WDR43-ALK* and *EML4-intergenic* fusion regions were visualized by the Integrative Genomic Viewer (IGV) software. **B** RNA sequencing reads indicating *EML4-ALK* fusion regions were visualized by the IGV software. **C** Possible schematic diagram of *WDR43-ALK* and *EML4-intergenic* fusions that were detected by DNA-based NGS, but a *EML4-ALK* fusion transcript was identified by RNA-based NGS. Red dashed lines indicate fusion breakpoints and red arrows indicate direction of transcription. **D** Positive ALK expression detected by IHC assay

Case 1, a representative intergenic complex rearrangement case, harbored *WDR43-ALK* (3′ intron1: 3′ intron19) and *EML4-intergenic* fusions identified by DNA-based NGS (Fig. 2A), with positive results detected using ALK IHC assay (Fig. 2D), but RNA-based NGS detected the canonical *EML4-ALK* fusion transcript joining *EML4* exon 13 to *ALK* exon 20 (Fig. 2B). Sequencing data indicated that the intergenic complex rearrangement involved multiple fusion junctions, comprising *EML4*, *LINC01913* upstream intergenic region, *WDR43* and *ALK* (Fig. 2C). Evidences of such intergenic complex rearrangements were also detected in case 2 and case 3 by DNA-based NGS, which harbored a canonical *EML4-ALK* variant 3 (E6:A20) transcript identified by RNA sequencing, and were positive by IHC and FISH assays (Table 2).

**Fig. 3:**
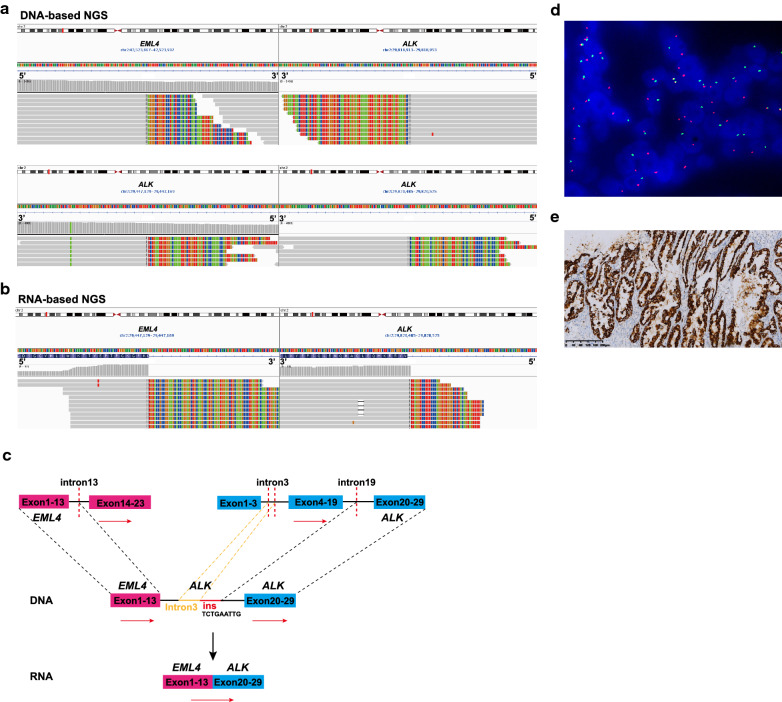
Representative example of intragenic complex rearrangements generating *EML4-ALK* fusion transcripts from case 4. **A** DNA sequencing reads indicating *EML4* (intron13)-*ALK* (intron3) fusion and *ALK* (intron19)*-ALK* (intron3) fusion regions were visualized by the IGV software. **B** RNA sequencing reads indicating *EML4-ALK* fusion regions were visualized by the IGV software. **C** Possible schematic diagram of genomic rearrangements involving the fusion breakpoints in DNA and RNA level. DNA-based NGS detected that intron 13 of *EML4* fused with intron 3 of *ALK*, and intron 3 of *ALK* jointed to intron 19 of *ALK* with a 9-bp insertion. RNA-based NGS detected the canonical fusion transcript joining *EML4* exon 13 to *ALK* exon 20. Red dashed lines indicate fusion breakpoints and red arrows indicate direction of transcription. **D** Positive *ALK* FISH pattern with separate red and green signals. **E** Positive ALK expression detected by IHC assay

The more remarkable observation, shared by cases 4–8, was the rare and complicated intragenic rearrangement of *ALK* or *EML4* gene identified at DNA level. Case 4 typically harbored multiple distinct rearrangements involving *ALK* locus, consisting of 5′ *EML4* (intron 13) and 3′ *ALK* (intron 3) fusion, *ALK-ALK* fusion in which intron 3 of *ALK* was jointed to intron 19 of *ALK* with a 9-bp insertion, *GALM-*3′ *EML4* fusion, and 5′ *ALK-intergenic* fusion (Fig. 3A). Only the first two connecting fusion-oncogene-associated rearrangements appeared capable of producing a functional pathogenic fusion transcript joining *EML4* exon 13 to *ALK* exon 20 detected by RNA-based NGS data (Fig. 3B and C). The other two fusions without transcription product may be the reciprocal fusions. Meanwhile, clear split signals of *ALK* gene were detected by FISH using a break-apart probe kit (Fig. 3D), and IHC test of the surgically resected sample revealed a positive result (Fig. 3E). Similarly, case 6 harbored 5′ *EML4* (intron 6) and 3′ *ALK* (intron 4) fusion with a 39-bp insertion and *ALK-ALK* fusion in which intron 4 of *ALK* was jointed to intron 19 of *ALK* with a 57-bp insertion, indicating to product the canonical *EML4-ALK* variant 3 (E6:A20) transcript without enough specimens for validation assays. In case 5, a special inversion of *ALK* gene from intron 18 to intron 19 was detected, in which 3′ intron 18 of *ALK* was jointed to 3′ intron 19 of *ALK* and 5′ intron 19 of *ALK* was jointed to 5′ intron 6 of *EML4* and RNA-based NGS detected the canonical *EML4-ALK* variant 3 (E6:A20) transcript. Similarly, inversions of *EML4* intron 6 were identified in case 7 and 8, which also harbored the canonical *EML4-ALK* variant 3 (E6:A20) transcript and were IHC and FISH positive (Table 2).

**Fig. 4:**
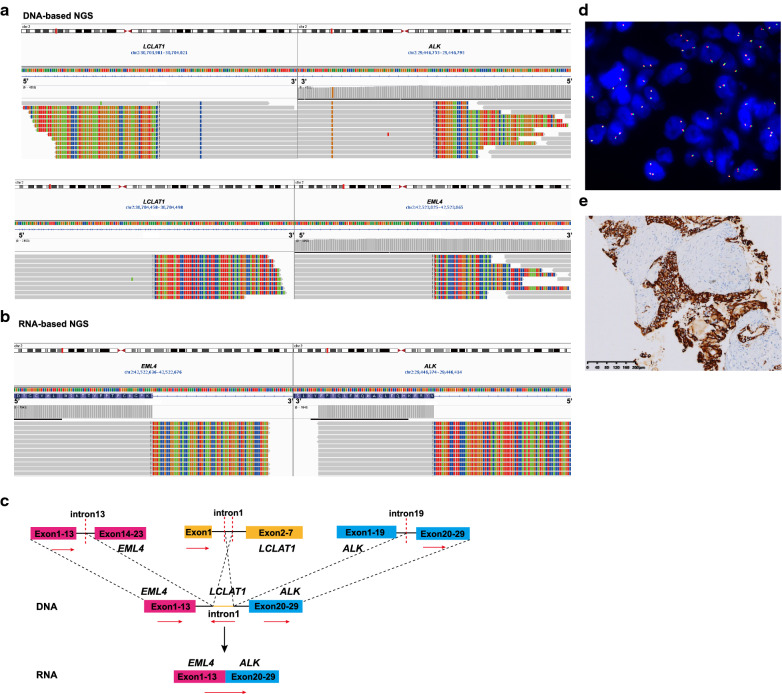
Representative example of “bridge joint” rearrangements generating *EML4-ALK* fusion transcripts from case 9. **A** DNA sequencing reads indicating *EML4-LCLAT1* and *LCLAT1-ALK* fusion regions were visualized by the IGV software. **B** RNA sequencing reads indicating *EML4* and *ALK* fusion regions were visualized by the IGV software. **C** Possible schematic diagram of *EML4-LCLAT1* and *LCLAT1-ALK* fusions that were detected by DNA-based NGS, but a *EML4-ALK* fusion transcript was identified by RNA-based NGS. Red dashed lines indicate fusion breakpoints and red arrows indicate direction of transcription. **D** Negative *ALK* FISH pattern with combined red and green signals. **E** Positive ALK expression detected by IHC assay

In cases 9–13, multiple gene fusions were identified, herein defined as “bridge joint” rearrangements owing to that both *EML4* and *ALK* jointed with an identical gene at the genomic level, respectively. Taking case 9 for example, DNA-based NGS detected that the intron 13 of *EML4* fused with the downstream region of intron 1 of *LCLAT1*, and the upstream region of intron 1 of *LCLAT1* joined to the intron 19 of *ALK* (Fig. 4A and C). Due to intronic splicing, it is reasonable that RNA-based NGS identified the canonical *EML4-ALK* variant 1 (E13:A20) transcript without intron1 of *LCLAT1* (Fig. 4B and C). IHC assays showed clearly positive ALK protein expression, but FISH revealed negative results, perhaps due to break-apart probe design or technical aspects yielding a risk of false-negative result (Fig. 4D and E) [20]. Similarly, in cases 10–13, DNA-based NGS revealed that the intron of *EML4* fusion partner gene firstly joined to the intronic region of a novel “bridge” gene and then to the intron of *ALK* kinase gene, as “bridge joint” complex rearrangements. Most of the intronic regions of the novel “bridge” genes were removed by splicing, leading to canonical *EML4-ALK* transcripts. In particular, the exon6 of “bridge” gene (*RUNX1*) was involved in the complex rearrangement and the *RUNX1-ALK* (exon6: exon20) transcript was detected in case 12, which may be part of the *EML4-RUNX1-ALK* (exon5: exon6: exon20) transcript hardly to be identified. Moreover, the *EML4- ALK* (exon5: exon20) transcript was also detected in case 12, perhaps due to the alternative splicing. Interestingly, case 14, harboring *EML4-RPIA* and *MAP4K3-ALK* fusions, was identified as the canonical *EML4-ALK* variant 1 (E13:A20) transcript, suggesting that *RPIA* and *MAP4K3* were both the “bridge” genes and their intronic regions were connected together (Table 2).

### Targeted therapies and clinical outcomes of complex *ALK* rearrangements

Among the 14 cases with complex *ALK* rearrangements, only 8 patients received targeted *ALK* inhibitors (crizotinib or alectinib) treatment, including 2 intergenic complex rearrangements, 3 intragenic complex rearrangements and 3 “bridge joint” rearrangements. Treatment and response to therapy, as defined by RECIST v1.1, were outlined in Table 3, which showed that 6 patients (75%) achieved clinical objective response, including 5 partial responses (PR) and 1 complete response (CR).

**Table 3:** Clinical outcomes in patients with complex *ALK* rearrangements who received targeted therapy

Case	DNA-based NGS	RNA-based NGS	IHC	FISH	Other variants	Matched treatment	Optimal response
1	*EML4-intergenic* (intron13: intergenic) *WDR43-ALK* (3′ intron1: 3′ intron19)	*EML4-ALK* (exon13: exon20)	ALK+	N/A	None	Crizotinib	PR
3	*EML4-intergenic* (intron6: intergenic) *PLXNA4-ALK* (3′ intron3: 3′ intron19) *intergenic-EML4* (intergenic: intron6)	*EML4-ALK* (exon6: exon20)	ALK+	+	*ALK* p.T1012M *ROS1* p.E1902K *TP53* p.R337C *TSC2* p.A678T	Crizotinib	PR
5	*EML4-ALK* (5′ intron6: 5′ intron19) *ALK-ALK* (3′ intron18: 3′ intron19) *IL1RAPL2-ALK* (5′ intron6: 5′ intron18) *ALK-PTCHD1* (3′ intron18: 3′ intron1)	*EML4-ALK* (exon6: exon20)	ALK+	+	*GNAS* p.A175T *TP53* splicing pathogenic	Alectinib	CR
6	*EML4-ALK* (intron6ins39: intron4) *ALK-ALK* (intron4ins57: intron19)	N/A	N/A	N/A	*ROS1* p.E1902K *SMAD4* p.N129H	Crizotinib	PR
8	*EML4-EML4* (5′ intron6: 5′ intron6) *EML4-ALK* (3′ intron6ins4: 3′ intron19)	*EML4-ALK* (exon6: exon20)	ALK+	+	*TP53* p.R273C *NOTCH3* p.A38Lfs	Crizotinib	PD
10	*EML4-NEB* (intron13: intron24) *NEB-ALK* (intron24: intron19)	*EML4-ALK* (exon13: exon20)	ALK+	+	None	Crizotinib	PR
13	*EML4-ZNF362* (intron13: intron1) *ZNF362-ALK* (intron1: intron19)	*EML4-ALK* (exon13: exon20)	ALK+	N/A	None	Alectinib	PR
14	*EML4-RPIA* (intron13: intron3) *MAP4K3-ALK* (intron2: intron19)	*EML4-ALK* (exon13: exon20)	ALK+	+	None	Crizotinib	SD

N/A: not available; +: positive results; −: negative results; CR: complete response; PR: partial response; SD: stable disease; PD: progression disease

Case 1 and case 3 with intergenic complex rearrangements both had positive response to crizotinib, and the endpoint of progression-free survival (PFS) was still not reached, lasted for at least 8 and 7 months, respectively (Table 3). Differential *ALK* inhibitor responses were observed among intragenic rearrangements variants in *ALK*-positive lung adenocarcinoma (case 5, case 6 and case 8). The identical *EML4-ALK* fusion cases 5 and 8, both got transcript joining *EML4* exon 6 to *ALK* exon 20 and positive results in FISH and IHC, achieved quite discrepant clinical outcome to crizotinib, CR for case 5 and progressive disease (PD) after 5 months treatment for case 8. We speculated that the other variant occurred in case 8, *TP53* p.R273C mutation, enhanced cancer cell proliferation, invasion and drug resistance [21]. As to the “bridge joint” rearrangements, one of the three cases, case 14, exhibited stable disease (SD) 4 weeks after crizotinib treatment (Table 3), different with the PR states of the other two cases, which implied that the poor clinical outcomes for *ALK* inhibitor in some patients could be caused by primary drug resistance to targeted therapies [22].

**Fig. 5:**
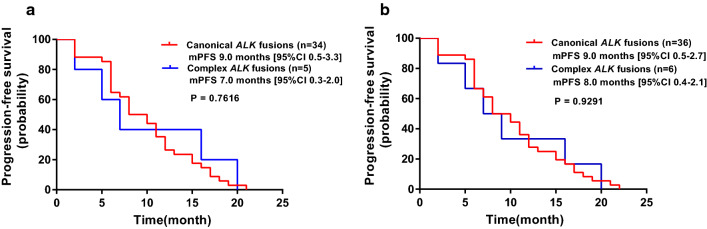
Survival curves for complex *ALK* fusion subtype and canonical *ALK* fusion subtype detected by DNA-based NGS. **A** Survival curves for patients with crizotinib treatment with complex *ALK* fusions (n = 5) and canonical *ALK* fusions (n = 34). **B** Survival curves for patients with crizotinib or alectinib treatment with complex *ALK* fusions (n = 6) and canonical *ALK* fusions (n = 36)

Patients with complex *ALK* fusions (n = 5) received crizotinib treatment exhibited comparable median progression-free survival (mPFS) with patients harboring canonical *ALK* fusions (n = 34), which displayed in Fig. 5A with the values of 7.0 months (95% CI 0.3–2.0) versus 9.0 months (95% CI 0.5–3.3) and the P value of 0.7616. Similarly, no significant difference in mPFS was observed between complex and canonical *ALK* fusions when patients with alectinib and crizotinib treatment were analyzed together (8.0 months [95% CI 0.4–2.1] versus 9.0 months [95% CI 0.5–2.7], P = 0.9291, Fig. 5B).

## Discussion

In this study, we identified three types of complex *ALK* rearrangements, intergenic complex rearrangements, intragenic complex rearrangements and “bridge joint” rearrangements. The complex *ALK* rearrangements could be attributed to a distinct mechanism, termed chromothripsis, happened at least 2 to 3% of all cancers and often promoted tumorigenesis in a wide variety of tumors [23]. Figures 2, 3 and 4 showed a possible mutational process mediated by inversion and chromothripsis. It caused a one-off chromosome breakage and subsequent random reassembly of the chromosome fragments, resulting in *ALK* and *EML4* joining to the intergenic/intragenic/ “bridge joint” regions respectively which were removed during transcription and generating the canonical *EML4-ALK* oncogenic fusions. *ALK* break-apart FISH analysis showed that there was more aberrant chromosome 2 fragmented and scattered in tumor cells, probably because chromosome structure was damaged severely by chromothripsis (Fig. 2D). Besides chromothripsis, translocation was reported as a novel mechanism of intragenic *ALK* rearrangements in neuroblastoma tumors in 2014 [24]. Another recent study revealed that intragenic complex rearrangements were related to *RB1* inactivation in *EGFR*-mutant lung cancer cell [25]. Here, we detected 5 cases intragenic complex rearrangement in lung adenocarcinoma, including 3 intragenic *ALK* rearrangements and 2 intragenic *EML4* rearrangements, which all generated canonical *EML4-ALK* fusion transcripts except for one not performed RNA-based NGS testing owing to insufficient specimen.

NGS technology has been widely used in rearrangements detection first in DNA-based method, which becomes a first-line pathological methodology in high-income country, such as US. Sampling requirement and quality metrics of DNA-based NGS is not as strict as RNA-based approach. One case in our study was not successfully performed RNA-based NGS because specimen cannot meet RNA stricter quality standards. DNA-based NGS can identify genomics rearrangements not limited to fusion, such as amplification of the *ALK* locus, which reveals a novel truncated form and activates drivers but not lead to fusion transcripts and proteins [26]. The targeted-capture DNA-based NGS panel are usually designed to target exonic and selected intronic regions of kinase genes, which has high probability to harbor the genomic breakpoints and could effectively identify kinase fusions. However, DNA-based NGS has some inherent limitations when targeted-capture introns are too long, or contain repetitive elements or involve complex genomic events [27]. The genomic rearrangement couldn’t be fully captured by DNA-based NGS panel when oncogenic fusion is caused by one or more complex DNA rearrangements. In contrast, RNA-based NGS offers a more direct approach to detect clinically actionable fusions, as RNA sequencing focused on exons post-splicing which may bypass genomic complexities [27]. As currently the most comprehensive and efficient strategy for exact fusion transcripts detection, RNA-based NGS testing is widely applied in the molecular diagnosis of gene fusion [28]. In our cohort, complex *ALK* rearrangements expressing canonical *EML4-ALK* fusion transcripts had been detected in 13 cases by DNA and RNA-based NGS. Genomic breakpoints within intronic regions of *EML4* were involved in the complex *ALK* rearrangements, which hardly detected by common NGS panel without probes capturing *EML4* introns. Using optimized probes tilling the selected intronic regions of *EML4*, genomic breakpoints within intronic regions of *EML4* were detected clearly by DNA-based NGS and illuminated the whole possible structures of the complex *ALK* rearrangements. Our finding suggested that it may be critical to utilize DNA-based NGS with optimized probes tilling the selected intronic regions of fusion partners followed by RNA-based NGS, which could effectively identify accurate oncogenic rearrangements and comprehensively guide optimal treatment decision not just in lung cancer but also across different types of tumor. Furthermore, there were 2 patient samples (case 9 and case 12) with discordant results between FISH and other assays. The discrepancy between multiple molecular testing could be considered as a ‘wake-up call’ for oncologists to ensure more accurate molecular diagnosis by identifying and functionally validating the clinically relevant complex genomic rearrangements.

Crizotinib, an oral small-molecule tyrosine kinase inhibitor (TKI) targeting *ALK*, *MET*, and *ROS1* tyrosine kinases, has been approved for *ALK*-rearranged NSCLC in USA, European Union, China and other countries, with objective response rate (ORR) of proximately 60.8% and median progression-free survival (mPFS) of 9.7 months [29]. Besides crizotinib, multiple second-generation (such as alectinib and ceritinib) ALK-TKIs have been developed for patients with *ALK*-positive NSCLC, all with higher potency than crizotinib [30–33]. Although ALK-TKI has dramatically expanded the therapeutic landscape of *ALK*-positive NSCLC, the substantial question, whether patients harboring complex genomic rearrangements could benefit from this target therapy, is not fully defined. Kodama et al. confirmed that alectinib and crizotinib were both effective against *EML4-ALK*-positive tumors mediated by chromothripsis in a patient-derived cell line, and the potency of alectinib was approximately 13-fold higher than crizotinib [23]. In our follow-up clinical data, 8 cases harboring complex *ALK* rearrangements showed the optimal responses with 1 CR (alectinib treatment), 5 PR (1 alectinib treatment and 4 crizotinib treatment), 1 SD (crizotinib treatment), and 1 PD (crizotinib treatment). It seemed that alectinib had a more remarkable response to complex *ALK* rearrangements than crizotinib in this “real world” data set. However, more studies should be performed in the future to verify the results with larger cohorts.

It was interested that case 8 achieved quite discrepant clinical outcome to crizotinib compared with case 5, PD after 5 months treatment for case 8 vs CR for case 5. Both patients had the identical rare and complicated intragenic *EML4-ALK* rearrangement detected in DNA and RNA-based NGS, and positive in FISH and IHC. The possible reason for the poor outcome might be the *TP53* p.R273C mutation detected in DNA-based NGS, which have been reported to enhance cancer cell proliferation, invasion and drug resistance [21]. It was reasonable that no significant difference was found in mPFS between the patients carrying complex and canonical *ALK* fusions regardless of their first-line treatment, crizotinib or alectinib, as all of them generated canonical *EML4-ALK* transcripts in RNA level. Limitation of the survival analysis in this study includes that the number of cases with complex *ALK* fusions receiving targeted therapy is relatively small (Additional file 1).

## Conclusions

This study firstly reveals the molecular characteristics and clinical outcomes of complex *ALK* rearrangements in NSCLC, sensitive to ALK inhibitors treatment, and highlights the importance of optimizing probe design of NGS panel for tilling the selected intronic regions of fusion partner genes. The discordant results of complex *ALK* rearrangements between DNA and RNA-based NGS indicate that DNA and RNA-based NGS assay should be both warranted in fusion detection. RNA and protein level assay may be critical in validating the function of complex *ALK* rearrangements in clinical practice for optimal treatment decision.

## Supplementary Information

**Additional file 1: Table S1.** The list of genes in DNA-based NGS panel.

## Data Availability

The datasets used and/or analysed during the current study are available from the corresponding author on reasonable request.

## Abbreviations

ALK: Anaplastic lymphoma kinase
EML4: Echinoderm microtubule-associated protein like-4
FFPE: Formalin-fixed paraffin embedded
FISH: Fluorescence in situ hybridization
IHC: Immunohistochemistry
NGS: Next-generation sequencing
NSCLC: Non-small cell lung cancer
PFS: Progression-free survival
TKI: Tyrosine kinase inhibitor
