# Supplementary material for: Molecular characteristics and clinical outcomes of complex ALK rearrangements identified by next-generation sequencing in non-small cell lung cancers
Source: J Transl Med. 2021 Jul 16;19:308. doi: 10.1186/s12967-021-02982-4 (PMC8283930; doi:10.1186/s12967-021-02982-4)
Supplement: Supplementary file 1 — Additional file 1: Table S1. The list of genes in DNA-based NGS panel. [file 12967_2021_2982_MOESM1_ESM.docx]

| Table S1. The list of genes in DNA-based NGS panel. | | | | |
| --- | --- | --- | --- | --- |
| **Fusions** | | | | |
| *ALK* | *BRAF* | *EGFR* | *FGFR2* | *FGFR3* |
| *JAK2* | *NRG1* | *NTRK1* | *NTRK2* | *NTRK3* |
| *PDGFRA* | *PDGFRB* | *RAF1* | *RET* | *ROS1* |
| **CNV** | | | | |
| *AKT1* | *ALK* | *AR* | *BRAF* | *BRCA1* |
| *BRCA2* | *CCND1* | *CD274* | *CDK12* | *CDK4* |
| *CDK6* | *DDR2* | *EGFR* | *ERBB2* | *ERBB3* |
| *ESR1* | *EZH2* | *FGFR1* | *FGFR2* | *FGFR3* |
| *FLT3* | *HRAS* | *JAK2* | *KIT* | *KRAS* |
| *MAP2K1* | *MAPK1* | *MDM2* | *MET* | *MYC* |
| *MYCN* | *NRAS* | *PDCD1LG2* | *PDGFRA* | *PDGFRB* |
| *PIK3CA* | *RAF1* | *RHOA* | *ROS1* | *SMO* |
| *VEGFA* |  |  |  |  |
| **SNV/Indel** | | | | |
| *ABL1* | *AKT1* | *ALK* | *APC* | *AR* |
| *ARAF* | *ARID1A* | *ATM* | *BCL2L11* | *BRAF* |
| *BRCA1* | *BRCA2* | *BTK* | *CCND1* | *CD274* |
| *CDH1* | *CDK12* | *CDK4* | *CDK6* | *CDKN2A* |
| *CTNNB1* | *DDR2* | *EGFR* | *EPCAM* | *ERBB2* |
| *ERBB3* | *ESR1* | *EZH2* | *FGFR1* | *FGFR2* |
| *FGFR3* | *FLT3* | *GNA11* | *GNAQ* | *GNAS* |
| *HRAS* | *IDH1* | *IDH2* | *JAK1* | *JAK2* |
| *JAK3* | *KIT* | *KRAS* | *MAP2K1* | *MAP2K2* |
| *MAPK1* | *MDM2* | *MET* | *MLH1* | *MPL* |
| *MSH2* | *MSH6* | *MTOR* | *MYC* | *MYCN* |
| *NF1* | *NF2* | *NFE2L2* | *NRAS* | *NTRK1* |
| *NTRK2* | *NTRK3* | *PDCD1LG2* | *PDGFRA* | *PDGFRB* |
| *PIK3CA* | *PMS2* | *PTCH1* | *PTEN* | *PTPN11* |
| *RAF1* | *RB1* | *RET* | *RHOA* | *ROS1* |
| *SMAD4* | *SMARCB1* | *SMO* | *STK11* | *TERT* |
| *TP53* | *TSC1* | *TSC2* | *VEGFA* | *VHL* |
| CNV, copy number variation; SNV, single nucleotide variant. | | | | |
